# Enhancing Specific-Antibody Production to the *ragB* Vaccine with *GITRL* That Expand Tfh, IFN-γ^+^ T Cells and Attenuates *Porphyromonas gingivalis* Infection in Mice

**DOI:** 10.1371/journal.pone.0059604

**Published:** 2013-04-01

**Authors:** Dong Zheng, Qiang Sun, Zhaoliang Su, Fanzhi Kong, Xiaoju Shi, Jia Tong, Pei Shen, Tianqing Peng, Shengjun Wang, Huaxi Xu

**Affiliations:** 1 Department of Immunology, Institute of Laboratory Medicine, Jiangsu University, Zhenjiang, PR China; 2 Affiliated People’s Hospital of Jiangsu University, Zhenjiang, PR China; 3 Department of Microbiology, Medway School of Pharmacy, University of Kent, Kent, United Kingdom; 4 Critical Illness Research, Lawson Health Research Institute, University of Western Ontario, London, Ontario, Canada; Nanyang Technological University, Singapore

## Abstract

The outer membrane protein RagB is one of the major virulence factors of the periodontal pathogen *Porphyromonas gingivalis* (*P. gingivalis*). In order to induce protective immune response against *P. gingivalis* infection, an *mGITRL* gene-linked *ragB* DNA vaccine (pIRES*-ragB-mGITRL* ) was constructed. Six-week-old female BALB/c mice were immunized with pIRES*-ragB-mGITRL* through intramuscular injection and then challenged by subcutaneous injection in the abdomen with *P. gingivalis*. RagB-specific antibody-forming cells were evaluated by an Enzyme-linked immunosorbent spot, and specific antibody was determined by enzyme-linked immunosorbent assay. In addition, the frequencies of Tfh and IFN-γ^+^ T cells in spleen were measured using flow cytometer, and the levels of IL-21 and IFN-γ mRNA or proteins were detected by real time RT-PCR or ELISA. The data showed that the *mGITRL*-linked *ragB* DNA vaccine induced higher levels of RagB-specific IgG in serum and RagB-specific antibody-forming cells in spleen. The frequencies of Tfh and IFN-γ^+^ T cells were obviously expanded in mice immunized by pIRES*-ragB-mGITRL* compared with other groups (pIRES *or* pIRES*-ragB* ). The levels of Tfh and IFN-γ^+^ T cells associated cytokines were also significantly increased in pIRES-*ragB-mGITRL* group. Therefore, the mice immunized with *ragB* plus *mGITRL* showed the stronger resistant to *P. gingivalis* infection and a significant reduction of the lesion size caused by *P. gingivalis* infection comparing with other groups. Taken together, our findings demonstrated that intramuscular injection of DNA vaccine *ragB* together with *mGITRL* induced protective immune response dramatically by increasing Tfh and IFN-γ^+^ T cells and antibody production to *P. gingivalis*.

## Introduction

Periodontitis is a frequent oral disease, attributing to the infection of microorganism. Extensive studies have suggested that the periodontitis not only damages the periodontal tissue, but also is an important incentive of many systemic diseases, such as cardiovascular diseases [1], [2], aspiration pneumonitis [3] and diabetes [4], as well as chronic kidney disease [5]. Thus, the prevention of periodontitis may be helpful for reducing the occurrence of systemic diseases.

*Porphyromonas gingivalis*, a gram-negative anaerobic bacterium, is considered as a major pathogen associated with chronic periodontitis disease [6], [7], [8]. A lot of virulence factors were possessed by *P. gingivalis* [9], particularly the outer membrane protein RagB. Curtis et al confirmed that *rag* locus is the pathogenicity island gene of *P. gingivalis* [10], which contained *ragA* and *ragB* gene, encoding the major outer membrane RagA and RagB respectively [11]. Nevertheless, the virulence of *Porphyromonas gingivalis* was significantly reduced in a mouse model of soft tissue destruction after using *ragB*-deletion mutant, compared to wild-type strains [12], [13]. Previous study showed that RagB, a 55-kDa immunodominant antigen [13], [14], can induce anti-RagB antibody response and play an important role in host-bacterium interactions of periodontal disease [14]. These studies indicate that RagB may be considered as a potential candidate vaccine antigen for preventing *P. gingivalis* infection.

Vaccine is one of many extremely effective methods to prevent pathogen from invasion. Since plasmid DNA inducing specific antibody response was demonstrated in animal models [15], the third generation vaccine was investigated, i.e., nucleic acid vaccine or DNA vaccine. Nucleic acid vaccine can express the target gene of interest for a long time in the body and induce protective immune response. Because of distinct advantages of DNA vaccine, it would be a critical issue in the vaccine field. With suitable molecular adjuvant, an effective vaccine can improve the immunogenicity and enhance the immune response or change the type of immune response, as well as remove pathogenic microorganisms from invading the organism. A number of cytokines and co-stimulatory molecules have been successfully used as adjuvant for augmenting DNA vaccine potency, such as, IL-12, IL-15 and OX40L [16], [17], [18].

Co-stimulatory molecules, the second signal for the activation of naive T cells, can promote T cell division, survival, and effector function as adjuvant [19]. The co-stimulatory molecule Glucocorticoid-induced TNFR family-related receptor ligand (GITRL) belongs to the TNFRSF member and is mainly expressed by dendritic cell, B cell, macrophage and endothelial cell [20]. GITR/GITRL system can be used as the co-stimulatory molecules for the T cells activation, to assist TCR-CD3 in the stimulation of T cells proliferation. The interaction of GITR and GITRL can upregulate the expression of IL-2-Rα and promote IL-2, IFN-γ production, also enable rapid activation and proliferation of CD8^+^ T cells [21]. Direct transfection with GITRL or GITRL protein can also promote the proliferation of T cells to help the body remove infected pathogenic microorganisms and induce immune response to tumor [22]. Based on these previous researches, we employed GITRL as the adjuvant in DNA vaccine, which will have multiple benefits in anti-infection immunity.

In this study, an *mGITRL* gene-linked *ragB* DNA vaccine (pIRES*-ragB-mGITRL* ) was constructed and used to induce protective immune response against *P. gingivalis* infection in the mice model, and the lesion development was utilized to evaluate the efficacy of pIRES*-ragB-mGITRL* for preventing the mice from the challenge by *P. gingivalis*. The results indicated that pIRES*-ragB-mGITRL,* as a new *ragB* DNA vaccine, played an important role in resisting *P. gingivalis* infection. As a part of *ragB* DNA vaccine, mGITRL may contribute to the production of *P. gingivalis* specific antibody via increasing Tfh and IFN-γ^+^ T cell. Therefore, *ragB* plus *mGITRL* DNA vaccine may be a potential candidate vaccine in clinic therapy for *P. gingivalis* infection.

## Materials and Methods

### Mice

6 weeks old female BALB/c mice (Supplied by the experimental animal center of Yangzhou University ) were used. The mice were maintained under specific-pathogen-free conditions (Laminar flow room or laminar flow type clean room ) at the experimental facility of Jiangsu University. All animal experiments were approved by the Institutional Committee on the Use of Animals for Research and Teaching, Jiangsu University.

### Bacterial and Cell Line Culture

**Figure 1:**
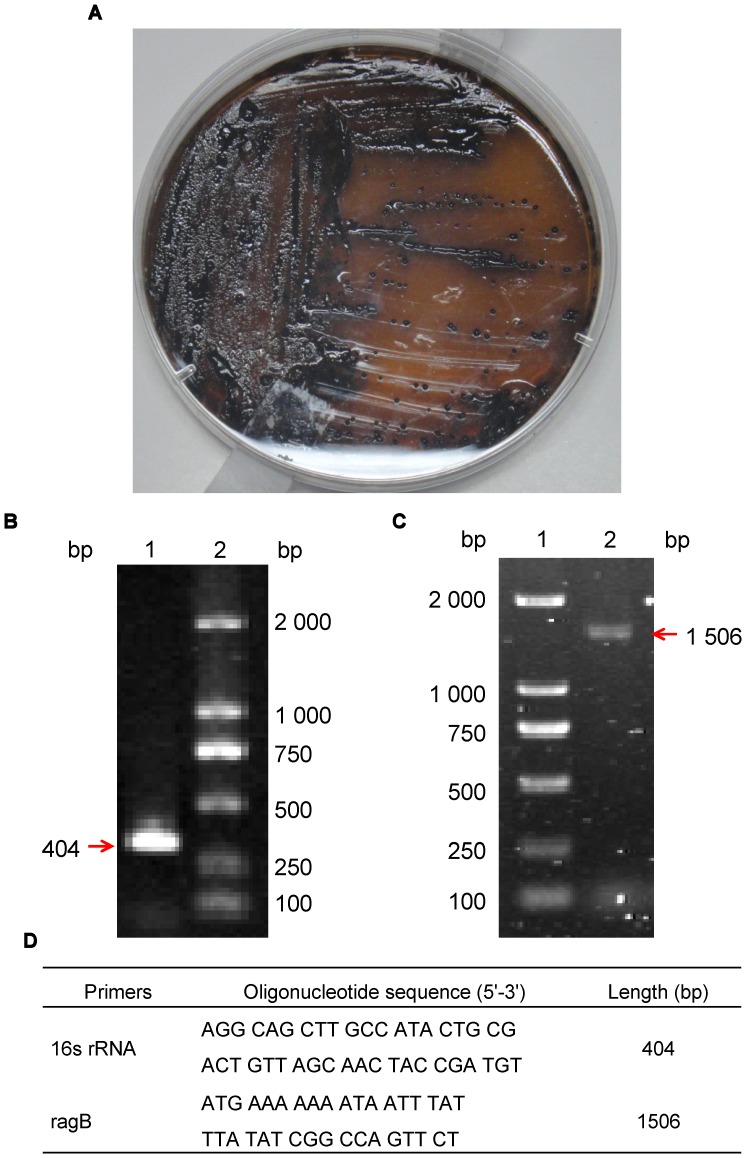
*P. gingivalis* strain W83 morphology and 16S rRNA gene analyzing. A, The colonial morphology of *P. gingivalis* strain W83 on the BHI agar plate. B, The PCR amplification result of 16s rRNA for *P. gingivalis*. Lane 1: The PCR amplification product; Lane 2: DL 2 000 DNA marker. C, The PCR amplification result of ragB from *P. gingivalis* strain W83. Lane 1: DL 2 000 DNA marker; Lane 2:The PCR amplification product. D, The oligonucleotide sequences of PCR primers for the genes of 16s rRNA and ragB from *P. gingivalis* strain W83.

*P. gingivalis* W83 strain [14] (Bought from Sichuan University) was grown on fastidious anaerobic agar plates with brain heart infusion (BHI; Tianhe, Hangzhou, China) supplemented with hemin (5 mg/L; Sanjie Biotechnology Co., Ltd., Shanghai, China), menadione (5 mg/L; Seventh Pharmaceutical Factory, Wuxi, China) and 5% sheep blood in anaerobic atmosphere containing 5% CO_2_, 5% H_2_, and 90% N_2_ for 72 h at 37°C [23]. *P. gingivalis* was confirmed via detecting its morphology and analyzing 16S rRNA genes with specific primers for PCR (Figure 1). The outer membrane protein of *P. gingivalis* W83, RagB, was expressed by our Lab [24]. COS-7 cell line cultured in DMEM medium supplemented with 10% FBS (GIBCO, UK).

### Construction of Recombined Plasmid

The region coding *ragB* domain of *P. gingivalis* W83 was amplified from plasmid pET-32a-*ragB* with specific primers [24]. The PCR products were digested with *Xho* I and *Mlu* I and ligated with pIRES (Invitrogen, USA) predigested with *Xho* I or *Mlu* I. The recombinant plasmid was named pIRES-*ragB*. The region coding *mGITRL* domain was amplified from plasmid pMD18-T-*mGITRL* with specific primers [25]. The PCR products were digested with *Xba* I and *Sal* I and ligated with pIRES-*ragB* predigested with *Xba* I and *Sal* I. The recombinant plasmid was named pIRES-*ragB-mGITRL*. Primers were as follows: ragB sense, 5'- CCGCTCGAGATGAAAAAAATAATTTAT-3' (*Xho* I), and antisense, 5'- TATACGCGTTTATATCGGCCAGTTCT-3' (*Mlu* I); mGITRL sense, 5'- ATCTCTAGAATGGAGGAAATGCCTTTG-3' (*Xba* I), and antisense, 5'-ATCGTCGACCTAAGAGATGAATGGTAG-3' (*Sal* I).

### Expression of Recombinant Plasmid in COS-7

pIRES, pIRES-*ragB*, or pIRES-*ragB-mGITRL* was transfected into COS-7 cells with LipofectamineTM 2000 (Invitrogen, USA). After forty-eight hours, cells were harvested and added 1× SDS- polyacrylamide gels loading buffer. The expression of *ragB* and/or *mGITRL* in transfected cells were analyzed by SDS- polyacrylamide gels and Western blot with anti-RagB (Prepared by our laboratory [24]) or anti-mGITRL antibody (Santa Cruz, USA).

### Mice Immunization

Five female BALB/c mice (6 weeks old) were used in each group, and immunized with pIRES-*ragB*, pIRES-*ragB-mGITRL*, pIRES or physiological saline via intramuscular injection (i.m.) at the quadriceps femoris of left thigh, respectively [26]. Prior to plasmid injection, each mouse was treated with 50 µl of 0.25% bupivacaine (Shanghai Zhaohui Pharmaceutical Co., Ltd., Shanghai, China) for three days consecutively [26]. For immunization, a total 50 µl physiological saline containing 50 µg plasmid DNA was injected into each mouse at the quadriceps femoris at day 0, 14 and 28. Blood samples were collected after mice were immunized at day 0 and 42. In order to detect RagB and mGITRL expression in immunized mice, the quadriceps femoris were isolated under euthanasia at day 42 after immunization and analyzed by SDS- polyacrylamide gels and Western blot with anti-RagB (Prepared by our laboratory [24]) or anti-mGITRL antibody (Santa Cruz, USA).

### Detection of RagB-specific Ab Production by ELISA

RagB-specific antibody in serum from immunizing mice was determined by ELISA. Briefly, polystyrene microtiter plates were coated with 1 µg/ml RagB in ELISA coating buffer at 4°C for overnight. Wells were blocked with 5% bovine serum albumin (JingKeHongDa Biotechnology Co., Ltd, Beijing, China) at 37°C for 2 hours. Duplicate serial twofold dilutions of samples in an appropriate range were added into the wells and incubated at 37°C for 1 hour. Each well was added with horseradish peroxidase-labeled goat anti-mouse immunoglobulin G (Wuhan Boster Bio-engineering Co., Ltd., Wuhan, China) and plate incubated at 37°C for 1 hour. The reaction was developed with 3, 3, 5, 5-tetramethyl-benzidine liquid substrate system (Sigma, American), which was stopped with 0.5 M H_2_SO_4_. Absorbance value at 450 nm was measured with a microplate reader (Bio-Rad, USA).

### Detection of Cells Producing Specific Antibody by ELISPOT

To assess numbers of RagB-specific antibody-forming cells at the single-cell level in spleen and bone marrow from immunizing mice, enzyme-linked immunospot (ELISPOT) assay was performed as previously described [27], [28]. Briefly, 96-well filtration plates with a nitrocellulose base (Millititer HA, Millipore, MA) were coated with 5 mg/ml RagB at 4°C for overnight [27]. Single-cell suspensions of spleen cells or bone marrow cells were added to wells at varied concentrations and incubated at 37°C for 5 hours in 5% CO_2_. After incubation and washing, horseradish peroxidase-labeled goat anti-mouse IgG was added and plate incubated at room temperature for 1 hours. The spots were developed with 3-amino-9-ethylcarbazole dissolved in 0.1 M sodium acetate buffer containing H_2_O_2_ at room temperature until seeing the spots. Numbers of RagB-specific antibody-forming cells were determined with a stereomicroscope (Olympus, Japan) and the results were given in 10^6^ spleen cells or bone marrow cells.

### Detection of Cytokines by Real Time RT-PCR and ELISA

To assess the level of cytokine mRNA (IL-21 and IFN-γ) in spleen, a standard real time RT-PCR amplification protocol was used. Briefly, total RNA was isolated from spleen cells with TRIzol reagent (Invitrogen, USA). Real time RT-PCR was conducted by the TAKARA SYBR Green mix (TAKARA, Japan) with Rotor-Gene 6000 (Qiagen, German) according to the manufacturer’s protocol. The gene expression was normalized against the house-keeping gene β-actin. Primers for target genes were designed with Oligo 6 online software and the sequences of primers were as follows: IL-21 sense, 5'-CCAGATCGCCTCCTGATTAG-3', and antisense, 5'-GAATCACAGGAAGGGCATTTAG-3'; IFN-γ sense, 5'-AAGCGTCATTGAATCACACC-3', and antisense, 5'- CGAATCAGCAGCGACTCCTTAG-3'; β-actin sense, 5'-TGGAATCCTGTGGCATCCATGAAAC, and antisense, 5'-TAAAACGCAGCTCAGTAACAGTCCG-3'.

Serum samples were analyzed for cytokines. IL-21 and IFN-γ were respectively quantified by ELISA kit (R&D, USA).

### Flow Cytometric Analysis for Tfh and IFN-γ^+^ T Cells

Single spleen cell suspensions from immunizing mice were gained by grinding with a sterile plunger of syringe and filtering with 70 mm cell strainers (BD Falcon, USA). For surface markers, spleen cells were stained with APC- conjugated CD3, PE-conjugated CD4, FITC-conjugated CXCR5 and PE-Cy5-conjugated ICOS mAbs (eBioscience, USA). CD4^+^ CXCR5^+^ ICOS^high^ T cells were defined as Tfh cells. For intracellular cytokine staining, spleen cells were stimulated with PMA (Sigma-Aldrich, USA, 50 ng/ml), ionomycin (Enzo, Switzerland, 1 mg/ml) and monensin (Enzo, Switzerland, 2 mg/ml) for 5 hours. Then spleen cells were stained with PE-CY5-conjugated CD3 and PE-conjugated CD4 mAbs (eBioscience, USA), fixed, permeabilized and stained with APC- conjugated IFN-γ mAb (eBioscience, USA) according to the Intracellular Staining Kit (Invitrogen, USA) instructions. Isotype-matched Ab controls were used in all procedures. The stained spleen cells were analyzed with Flow Cytometer (Becton Dickinson, USA) and data was analyzed with WinMDI 2.9 software.

### Induction of Murine Lesion Model

To examine the protective effect of pIRES*- ragB* and pIRES*- ragB- mGITRL* DNA vaccine, female BALB/c mice were challenged with *P. gingivalis* strain W83 after immunizing for 42 days [29], [30]. Briefly, *P. gingivalis* W83 was cultured at anaerobic condition and harvested at logarithmic growth phase, washed three times with sterile PBS, and resuspended in sterile PBS. *P. gingivalis* W83 was quantified via culturing on the BHI agar plate with ten-fold dilution serial. At the 42nd day after first time immunization, each mouse was challenged with 1×10^9^ cells of *P.gingivalis* W83 by subcutaneous injection (100 ul) in the abdomen [29], [30]. The lesion sizes of mice were measured at 48 hours after challenging, and the samples (serum, spleen cells and bone marrow cells) were collected for following experiment.

### Statistical Analysis

All data were summarized as means ± SD. The significance of difference between two groups was evaluated by the unpaired Student’s t-test. Differences in multigroup comparisons were performed with one-way ANOVA followed by Newman-Keuls test. All conclusions were based on significance levels of P<0.05.

## Results

### Construction of Recombinant DNA Vaccine pIRES-ragB and pIRES-ragB-mGITRL

**Figure 2:**
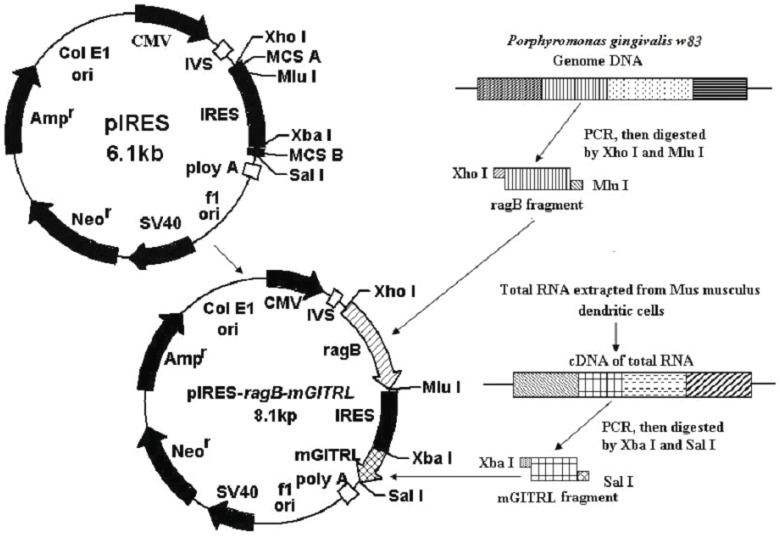
Construction of recombinant plasmid pIRES*-ragB-mGITRL* for immunization. Amplified *ragB* and *mGITRL* genes were inserted into the eukaryotic expression vectors pIRES, respectively. CMV, human cytomegalovirus immediate-early promoter; IRES, internal ribosome entry site; SV40, simian virus 40; Amp^r^, ampicillin resistance gene; Neo^r^, neomycin resistance gene.

The whole construction process of DNA vaccine was showed in Figure 2. The *ragB*-carrying DNA fragments were amplified from plasmid pET-32a-*ragB* by PCR. The PCR products were digested with double restriction endonuclease (*Xho* I and *Mlu* I) and ligated into pIRES predigested with *Xho* I or *Mlu* I. The obtained recombinant plasmid pIRES-*ragB* was confirmed with double restriction endonuclease digestion (Figure 2), PCR and DNA sequencing (data not shown). The construction of pIRES-*ragB-mGITRL* was similar as pIRES-*ragB*.

### Expression of RagB and mGITRL in vitro and in vivo

**Figure 3:**
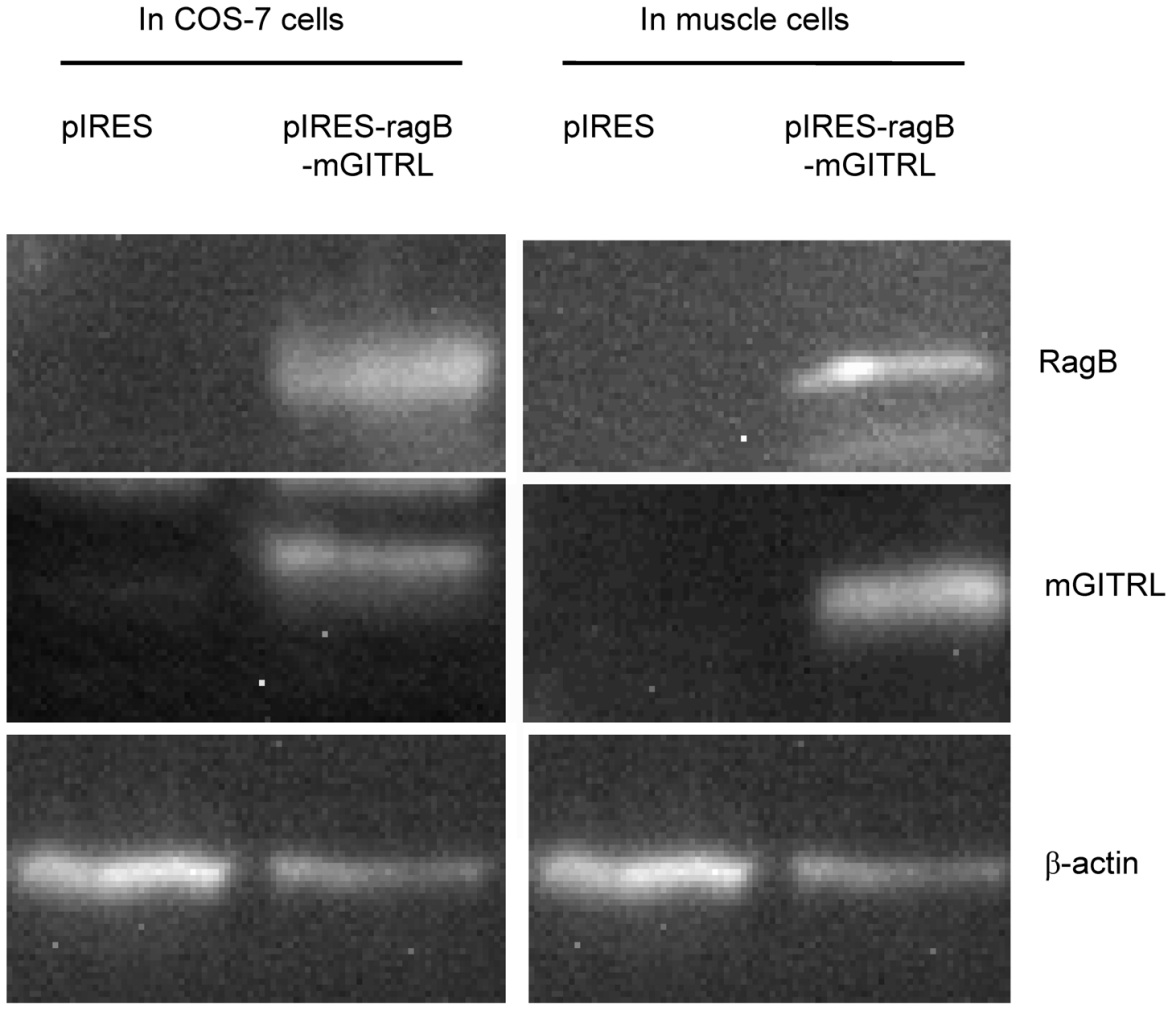
Expression of *RagB* or *mGITRL* in COS-7 cells or muscle cells of mice. These data were representative of three independent experiments with similar results.

To assess expression of *ragB* and *mGITRL* in vitro, we transfected the plasmids into COS-7 cells for 48 hours, and analyzed their protein expression with specific antibodies by western blot (Figure 3). The results showed the protein RagB (55-kDa) and mGITRL (20-kDa) levels were increased significantly in transfected COS-7 cells with interest plasmids, comparing with non-transfected or transfected with empty plasmid pIRES group.

Furthermore, in order to investigate the expression of *ragB* and *mGITRL* in vivo, DNA vaccines were injected into the BALB/c mice. After injection for 42 days, the muscles of injection site were isolated for the protein expression analysis by western blot (Figure 3). Similarly, we observed the enhanced expression of *ragB* and *mGITRL* in injected groups compared with control group. These data indicated that pIRES-*ragB* or pIRES*-ragB-mGITRL* could express recombinant proteins RagB and mGITRL in eukaryotic cells.

### *ragB* Plus *mGITRL* Elicited High-titers RagB-specific Ab Responses and Abundant RagB-specific Antibody-forming Cells

**Figure 4:**
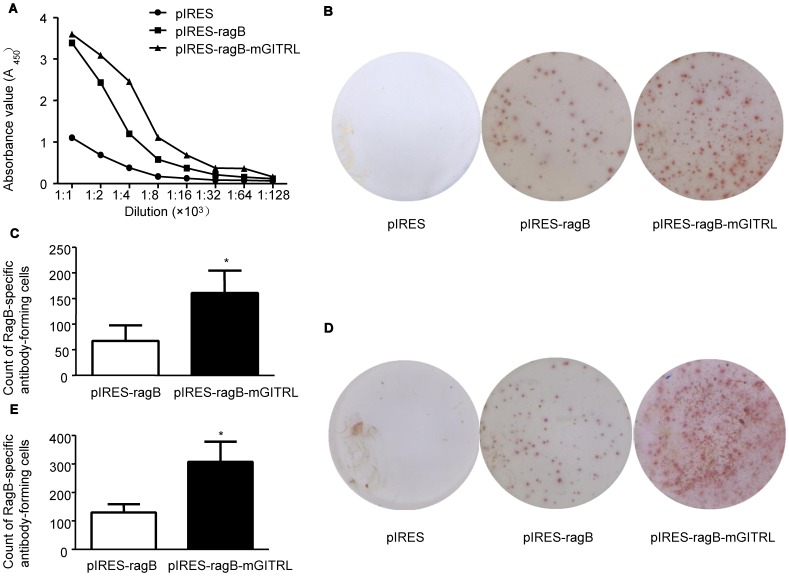
The anti-RagB IgG antibody levels in serum and RagB-specific antibody-forming cells in spleens and bone marrow. A, The levels of RagB-specific antibody in pIRES, pIRES-*ragB* and pIRES*-ragB-mGITRL* groups; The sum of RagB-specific antibody-forming cells of spleen (B) or bone marrow (D) in three group respectively. The comparison of RagB-specific antibody-forming cell level in spleen (C) or bone marrow (E) between pIRES-ragB-mGITRL group and pIRES-ragB group. The results are expressed as means ± SD obtained for five mice per group. *P<0.05 vs. pIRES-*ragB* group.

Six-week-old female BALB/c mice were immunized by intramuscular injection (i.m.) with plasmid pIRES, pIRES-*ragB* or pIRES*-ragB-mGITRL,* and then given twice booster immunization respectively at 14 and 28 days after the primary vaccination. RagB-specific antibodies in serum of mice were identified by ELISA (Figure 4A). The analysis of antibodies levels showed the pIRES-*ragB* group of mice produced higher levels of RagB-specific IgG antibody than the pIRES group. It is surprising that pIRES-*ragB-mGITRL* strongly elicited higher levels RagB-specific IgG antibody response than the pIRES-*ragB* group.

To further clarify the mechanism of RagB-specific Ab production, RagB-specific antibody-forming cells in spleen and bone marrow of mice were detected with ELISPOT (Figure 4B and 4D). The data suggested there were abundant RagB-specific antibody-forming cells in pIRES-*ragB-mGITRL* immunized mice. But only a number of RagB-specific antibody-forming cells were found in pIRES-*ragB* mice, and even less in the pIRES group. (Figure 4C and 4E).

### Intramuscular Injection of *mGITRL* Expanded Tfh and IFN-γ^+^ T Cell in Spleen

**Figure 5:**
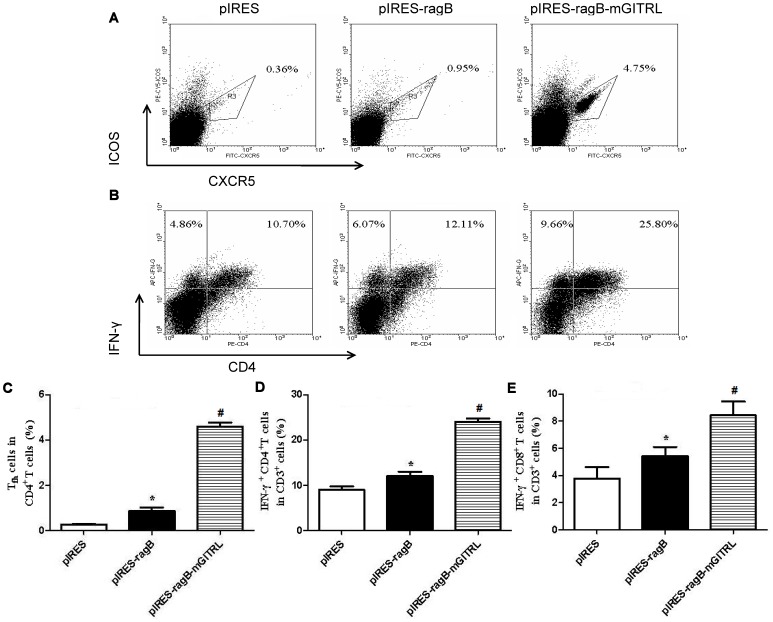
Tfh and IFNγ^+^-T cells were detected by FCM in spleens of mice. A and B showed the rate of Tfh (CD4^+^ CXCR5^+^ ICOS^high^ T cells ) in spleen of pIRES, pIRES-*ragB*, or pIRES-*ragB-mGITRL* group, respectively; C, D, E indicated the levels of Tfh, IFN-γ^+^ CD4^+^ T and IFN-γ^+^ CD8^+^ T cells from groups of pIRES, pIRES-*ragB* and pIRES-*ragB-mGITRL.* Isotype controls were used to determine the positive cells, and the values were gated on the CD3^+^ cells for IFN-γ^+^ CD4^+^ T or IFN-γ^+^ CD8^+^ T cells. The results are expressed as means ± SD obtained for five mice per group. *P<0.05 vs. pIRES group, #P<0.05 vs. pIRES-*ragB* group.

In order to clarify the possible reasons of higher-titers RagB-specific Ab responses in mice immunized by plasmid pIRES-*ragB-mGITRL*, the frequencies of Tfh cell and IFN-γ^+^ T cell in spleens was analyzed with flow cytometry. Surprisingly, Tfh cell in spleens of mice immunized with plasmid contained *mGITRL* gene were expanded by ten-fold than the mice immunized with non-*mGITRL* plasmid (Figure 5A and 5C). At the same time, IFN-γ^+^ T cell was also significantly up-regulated by plasmid pIRES-*ragB* and pIRES-*ragB-mGITRL* after challenged with *P. gingivalis* strain W83 (Figure 5B, 5D and 5E).

**Figure 6:**
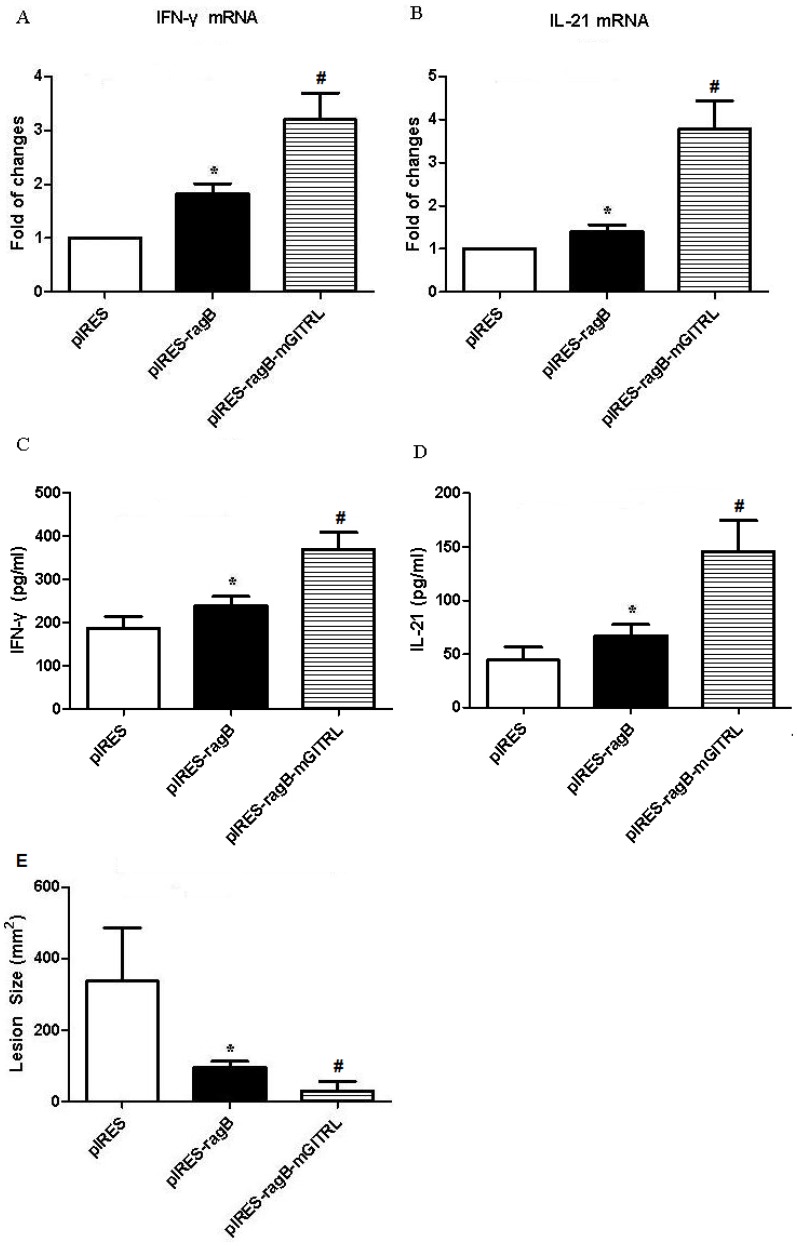
IFN-γ and IL-21 were up-regulated in serum of mice immunized with *RagB* plus *mGITRL*. The mRNA and protein levels of IFN-γ and IL-21 in the spleen of pIRES, pIRES-*ragB* and pIRES-*ragB-mGITRL* groups (A, B, C and D). The results were expressed as means ± SD obtained for five mice per group. *P<0.05 vs. pIRES group, #P<0.05 vs. pIRES-*ragB* group. E:Lesion area of mice challenged by *P. gingivalis* W83 strain. The results were expressed as means ± SD obtained for five mice per group. (*P<0.05 vs. pIRES group, #P<0.05 vs. pIRES-*ragB* group).

To further demonstrate the roles of *RagB*- and *mGITRL*-contained plasmid in anti-infection by challenging with *P. gingivalis*, we analyzed mRNA levels of IFN-γ and IL-21 in spleen cells of mice by real time RT-PCR, and their protein levels by ELISA. The results showed mRNA and protein levels of IFN-γ and IL-21 were both enhanced in pIRES-*ragB* group and pIRES-*ragB-mGITRL* group compared with pIRES group (Figure 6A, 6B, 6C and 6D). Obviously, pIRES-*ragB-mGITRL* group contained significantly higher concentrations of IFN-γ and IL-21 than the other two groups (Figure 6C and 6D). Taken together these data suggested that the spleen of mice immunized with DNA vaccine could be initiated with Th1 cell response and up-ragulated Tfh through administrating ectogenesis RagB and mGITRL.

### *pIRES-ragB-mGITRL* Reduced the Lesion of Mice Challenged by *P. gingivalis*

The key role of the vaccine is to protect the organism from being infected with pathogenic microorganisms. Therefore, we utilized a mouse lesion model to evaluate the protective effect of the plasmid pIRES-*ragB* and pIRES*-ragB-mGITRL* [31]. After *P. gingivalis* strain W83 infection, the mice of pIRES-*ragB-mGITRL* group showed much smaller lesions than other two groups (Figure 6E). Surprisingly, no lesion was found in two mice immunized with pIRES-*ragB-mGITRL*.

## Discussion

RagB, the major outer-membrane protein of *P. gingivalis*, is a 55-kDa immunodominant lipoprotein and an important pathogenic factor [9], [10], [11]. RagA, another major outer membrane protein, is a 115-kDa TonB-dependent outer membrane receptor [10]. RagA and RagB form a TonB-dependent outer membrane receptor complex, which help bacteria to acquire and transport macromolecule substance from the outer environment, and associate with the survival and dissemination of bacteria [10]. Furthermore, RagB protein can trigger the immune response and inflammation of host [32]. Previous studies showed that rag locus was a novel pathogenicity island of *P. gingivalis* [10], and RagB obviously damaged the periodontal soft tissue in a mouse model of infection. However, the virulence of *P. gingivalis* strain was attenuated by *ragB* deletion [13]. Meanwhile, the local injuries of the mice immunized by RagB protein were alleviated. With these findings, RagB protein may be a potential candidate vaccine for periodontal disease.

In the present study, eukaryotic expression vector pIRES was used to carry *ragB* gene for constructing a pIRES*-ragB*-*GITRL* plasmid, which was employed to induce protective immune response in mice by intramuscular injection. In addition, 0.25% bupivacaine was used prior to plasmid immunization for promoting plasmid absorption. The results of western blot from COS-7 cells or mice suggested the transfection of *ragB* plasmid in vitro or in vivo had high efficiency. These data collectively indicated that anti-RagB specific antibody responses were generated by intramuscular injection of pIRES*-ragB or* pIRES*-ragB*-*GITRL*. After challenged with *P. gingivalis* strain W83, the mice immunized by pIRES-*ragB or* pIRES*-ragB*-*GITRL* showed a decreased lesion size (70%) compared with control group. These results demonstrated the plasmid of pIRES*-ragB*-*GITRL* may be a new kind of DNA vaccine for effectively inducing protective immune response against *P. gingivalis* infection.

Along with the vaccine development, the researchers pay close attention to the adjuvant. As we known, B cells induce high-affinity antibody responses and B-cell memory needs the assistant of T cells. Recently, the studies have revealed that a discrete follicular population of T cells, i.e., Tfh, has a crucial role in this process [33], [34].

Tfh cells, defined with CD4^+^ CXCR5^+^ ICOS^high^ T cell, interact with and stimulate the generation of antigen-specific B cells, which classically occurs in germinal center (GC) located in the B cell follicles, accordingly regulating B-cell-mediated humoral immunity [34], [35], [36]. The interaction of Tfh and B cells promotes the selection and survival of high-affinity memory B cells [37]. The activation of Tfh cells highly express IL-21 [38], which is the most potent cytokine for driving plasma cell differentiation and promoting GC development and maturation [39]. Hong JJ et al study showed that Tfh cells interaction with B cells correlates with production of SIV-specific antibodies [40]. The surface of Tfh cells express glucocorticoid-induced TNFR-Related (GITR) protein, and GITR/GITRL system can expand the Tfh cell [34]. GITRL, the ligand of GITR, is primarily expressed on the surface of dendritic cells, macrophages, B cells and also epithelial cells [20], [41], [42]. GITR/GITRL system can activate T lymphocytes as the co-stimulatory molecule, stimulating the proliferation of T cells by promoting the action of TCR-CD3 [20]. Additionally, CD8^+^ T cells can be quickly activated and proliferated by this system. In our study, GITRL was used as an adjuvant, and linked to pIRES*-ragB*. We found that mGITRL protein could be effectively expressed in COS-7 cells and in mice. The proliferation of Tfh cells was enhanced in the spleen of mice immunized with pIRES*-ragB*-*GITRL*, and mRNA or protein levels of IL-21 were increased in spleens and serum of immunizing mice. Consistent with the previous studies, the Tfh cells activated and promoted B cells proliferation via secreting IL-21 and upregulated IL-21 receptor on B cells [43]. Moreover the analysis of RagB-specific antibody in serum of mice and the amount of RagB-specific antibody-forming cells in spleen and bone marrow of mice showed that they were significantly up-regulated for two or three fold, respectively. At the same time, the mice immunized with pIRES*-ragB*-*GITRL* were challenged with *P. gingivalis* strain W83 and had a decreased lesion size comparing with pIRES*-ragB* or control group. Therefore, our results suggested GITRL played a key role in the process of antigen-specific antibody production and anti-infective immunity.

Because of the GITR expression in T cells [44], GITRL may be acting in T cells to promote its activation and proliferation. The observation showed that the spleen or serum of mice immunized by *ragB* plus *mGITRL* produced high level of IFN-γ which, from IFN-γ^+^ T cells, enhanced the activation of Macrophage and NK cell for clearing bacteria [45], [46]. Moreover, IFN-γ^+^CD8^+^T cell may have direct response to pathogen or pathogen infected cells to promote clearing. Owing to the *GITRL* gene-linked *ragB* plasmid application, mice challenged with *P. gingivalis* W83 strain formed smaller lesion size than those in control group or only *ragB* plasmid administered group.

**Figure 7:**
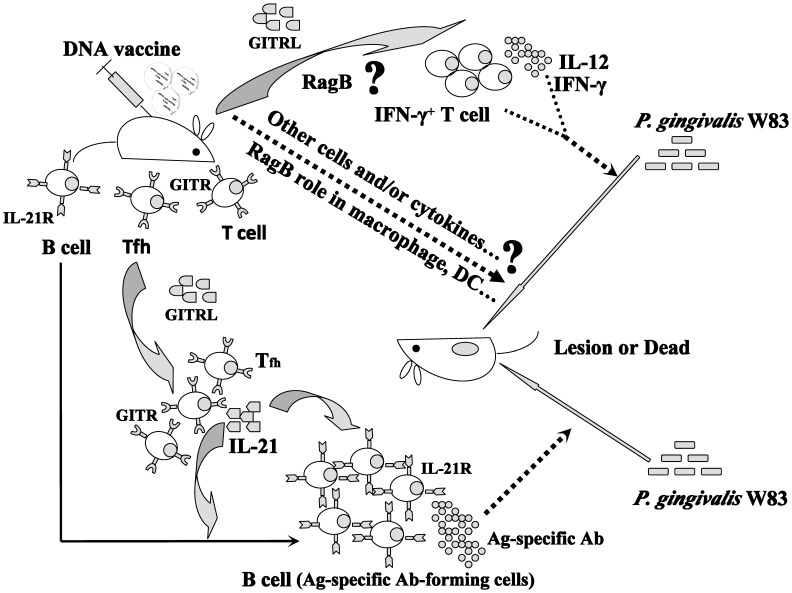
Model of *ragB* plus *mGITRL* DNA vaccine anti-infection by *P. gingivalis* W83. DNA vaccine, pIRES-*ragB* and pIRES-*ragB-mGITRL* injection enhanced the proliferation of Tfh and IFN-γ^+^ T cells by GITR/GITRL system, with inducing a large number of cytokines such as IFN-γ and IL-21, and thus promoted the production of RagB-specific antibody, protected mice from an attack of *P. gingivalis* W83.

In summary, our studies demonstrated pIRES-*ragB-mGITRL* injection in mice dramatically decreased the lesion size, and inhibited the development of infection by *P. gingivalis.* Further mechanisms studies revealed that pIRES-*ragB* or pIRES-*ragB-mGITRL* injection significantly enhanced the proliferation of Tfh and IFN-γ^+^ T cells by GITR/GITRL system, inducing a large number of cytokines such as IFN-γ and IL-21, and thus promoted the production of RagB-specific antibody. Our findings strongly indicated that pIRES-*ragB-mGITRL* may become a potential candidate of DNA vaccine for inhibiting *P. gingivalis* infection, and GITRL may be a new type of adjuvant (Figure 7). Further studies will focus on how RagB regulates cytokines production in mice, via lipid raft or the surface receptor of innate immunity cells, like the major outer membrane protein of a periodontopathogen, *Treponema lecithinolyticum* [47], and whether the action of RagB and mGITRL can be blocked by anti-serum from the mice injected with pIRES-*ragB-mGITRL*.
