# Correction: Enhancing Specific-Antibody Production to the *ragB* Vaccine with *GITRL* That Expand Tfh, IFN-γ^+^ T Cells and Attenuates *Porphyromonas gingivalis* Infection in Mice

**DOI:** 10.1371/journal.pone.0301151

**Published:** 2024-03-20

**Authors:** Dong Zheng, Qiang Sun, Zhaoliang Su, Fanzhi Kong, Xiaoju Shi, Jia Tong, Pei Shen, Tianqing Peng, Shengjun Wang, Huaxi Xu

This correction addresses an error in Fig 3 of [1].

Specifically, the incorrect panel was used for the Fig 3 In muscle cells β-actin results. The corrected Fig 3 is provided with this notice. The original data underlying the published results are no longer available.

The authors apologize for the error.

**Fig 3:**
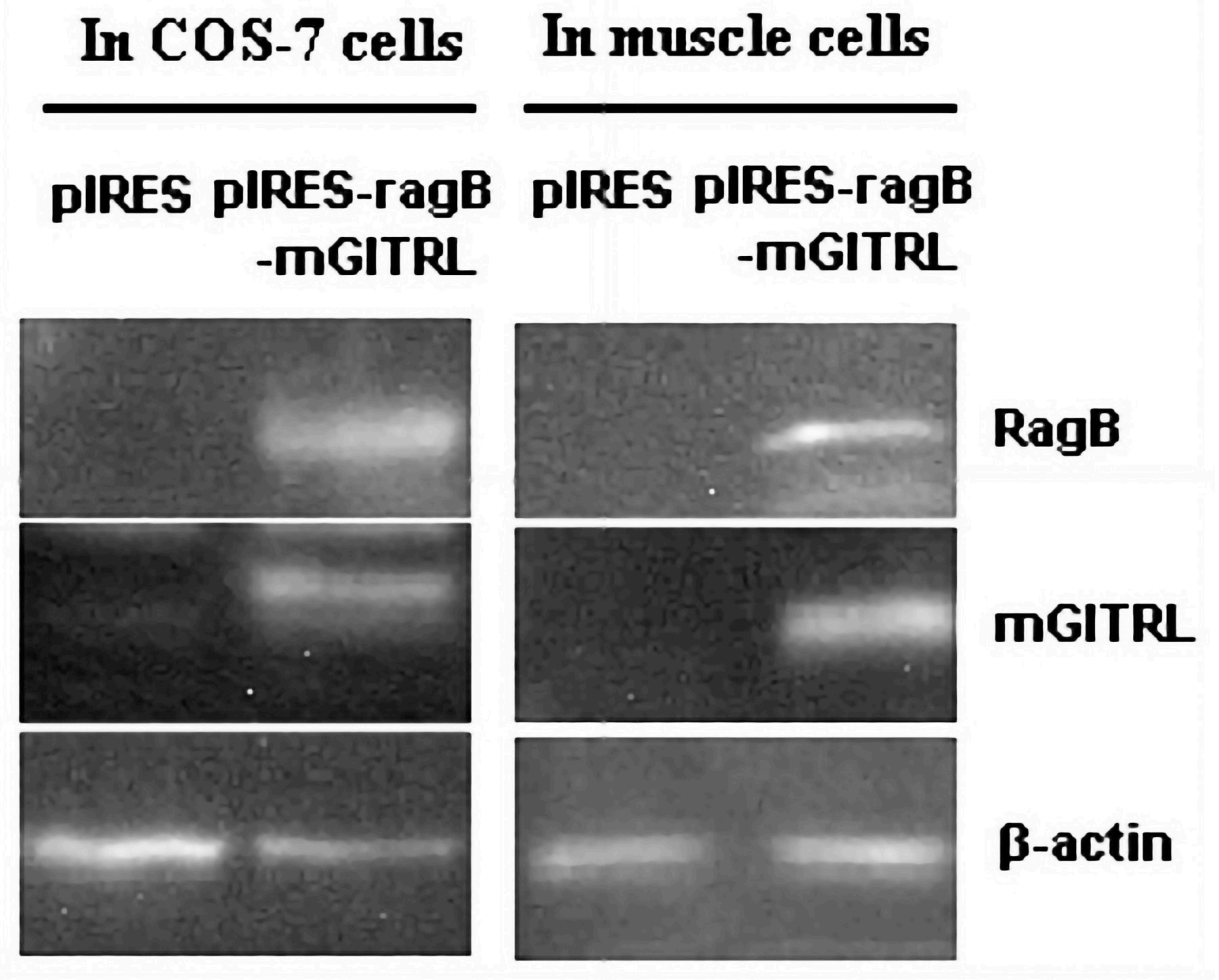
Expression of *RagB* or *mGITRL* in COS-7 cells or muscle cells of mice. These data were representative of three independent experiments with similar results.
